# Alexithymia and personality traits of patients with inflammatory bowel disease

**DOI:** 10.1038/srep41786

**Published:** 2017-02-02

**Authors:** D. La Barbera, B. Bonanno, M. V. Rumeo, V. Alabastro, M. Frenda, E. Massihnia, M. C. Morgante, L. Sideli, A. Craxì, M. Cappello, M. Tumminello, S. Miccichè, L. Nastri

**Affiliations:** 1 Department of Experimental Biomedicine and Clinical Neuroscience, Unit of Psychiatry, University Hospital, Palermo, Italy; 2 Unit of Nefrology II with Dialysis and Renal Transplantation, ARNAS Civico Di Cristina Benfratelli, Palermo, Italy; 3 Biomedical Department of Internal and Specialty Medicine, Regional Reference Center for Metabolism rare pathologies, University Hospital, Palermo, Italy; 4 Biomedical Department of Internal and Specialty Medicine, Unit of Gastroenterology, University Hospital, Palermo, Italy; 5 Department of Economics, Management and Statistics, University of Palermo, Palermo, Italy; 6 Department of Physics and Chemistry, University of Palermo, Palermo, Italy

## Abstract

Psychological factors, specific lifestyles and environmental stressors may influence etiopathogenesis and evolution of chronic diseases. We investigate the association between Chronic Inflammatory Bowel Diseases (IBD) and psychological dimensions such as personality traits, defence mechanisms, and Alexithymia, i.e. deficits of emotional awareness with inability to give a name to emotional states. We analyzed a survey of 100 patients with IBD and a control group of 66 healthy individuals. The survey involved filling out clinical and anamnestic forms and administering five psychological tests. These were then analyzed by using a network representation of the system by considering it as a bipartite network in which elements of one set are the 166 individuals, while the elements of the other set are the outcome of the survey. We then run an unsupervised community detection algorithm providing a partition of the 166 participants into clusters. That allowed us to determine a statistically significant association between psychological factors and IBD. We find clusters of patients characterized by high neuroticism, alexithymia, impulsivity and severe physical conditions and being of female gender. We therefore hypothesize that in a population of alexithymic patients, females are inclined to develop psychosomatic diseases like IBD while males might eventually develop behavioral disorders.

Chronic Inflammatory Bowel Diseases are chronic disorders with uncertain etiology, characterized by chronic and idiopathic inflammation of the bowel. It is possible to distinguish two types of diseases within this category: Crohn’s disease (CD) and Ulcerative Colitis (UC). Data of scientific literature show that psychological factors together with particular lifestyles and environmental stressors have an influence not only on etiopathogenesis, but also on relapses of such chronic diseases^1^,^2^.

For years Chronic Inflammatory Bowel Diseases (IBD) have been thought of as examples of psychosomatic disorders^3^. In fact, our theoretical background on patients affected by CD and UC refers to a model according to which the somatic pathology results from the altered relationship between the body and the external world and the possibility that the body (the “not symbolic system”) reacts to difficulties whenever the mind (i.e. the “symbolic system”) fails to do so properly^4^. The origins of this model track back to the studies of the French school of Marty and De M’Uzan^5^ who described the psychosomatic personality as characterized by deficits in the processing of emotions and by psychological trauma that are thus predominantly expressed in a somatic sense. While the neurotic patient would use psychic defenses that can help him to reduce the anxiety-inducing tension produced by unconscious conflicts, the psychosomatic patient would lack the capacity to symbolize, thus using its body for discharging internal tensions. In 1963 Marty and de M’Uzan delineated therefore the psychosomatic personality as characterized by an attitude of hyper-normality, with a conformist adaptation to the environment and social needs, and a particular cognitive style called “operational thinking” (*pensèe operatoire*)^6^. The operational thinking is characterized by a number of deficit. The external drivers can not be processed at a mental level and thus are discharged directly into the body. The operational thinking is outlined by the absence of dream-like ability, fantasy and representational skills. Individuals who have this type of thinking manifested poor verbal skills, very concrete thought and turned to everyday reality, without imagination and emotional investment^7^. Initially, seven diseases were spotted. They are now known as classic psychosomatic diseases: peptic ulcer, bronchial asthma, hypertension, thyrotoxicosis, ulcerative colitis, rheumatoid arthritis, neurodermatitis.

Marty and De M’Uzan’s observations allowed psychoanalysts Sifneos and Nemiah to study cognitive characteristics of patients affected by psychosomatic disorders. They found out that most patients had a clear difficulty to describe feelings together with a very poor phantasmatic activity^8^. After Sifneos, such a condition has been named as “Alexithymia”, which literally means “emotions without words”. These patients can experience emotions such as excitability, irritability and sadness, but cannot give them any meaning^9^. In general these are, apparently, well adapted people, usually have a job, a family, some friends; such relationships though are superficial or of great dependency, based on an attitude of “pseudo-normality”^10^,^11^, which, at a closer look, reveals a poor contact with emotional life. Alexithymia, thus, must not be considered as a diagnosis, but a stable personality trait that interacts with stressful events, aspecifically predisposing towards the development of psychosomatic disorders^12^,^13^.

In recent years the concept of psychosomatic disease is, however, being replaced by the concept of “multi-causal model” so that disease once called “psychosomatic” would arise due to the coexistence of multiple influences on health (with no distinction between physical and mental) of intra-physicic, relational and social situations, according to Engel’s biopsychosocial model^14^. In more general terms we turn away more and more from the idea of a specific personality of the psychosomatic patient, leading to a linear and uni-casual explanation of the disease. Nowadays, the pathology is seen as deriving from the lack of one or more mental functions, as in the case alexithymia, in addition to other factors such as: the relational conditions, the time of the life cycle, the vulnerability of a particular organ or system, the physical environment, the relationship with the therapist. The somatic symptom shows up whenever a mental representation of affection is lacking. The somatic symptom occurs just because the reaction to a situation of life has been unable to find the way of the psyche (the symbolic system).

Our study aims at evaluating the influence of different factors such as certain personality traits in the etiopathogenesis and development of inflammatory bowel diseases. In this respect, the role that stress plays in relation with the pathogenesis and course of chronic inflammatory bowel diseases (IBD) is of interest to researchers^15^. In the largest part of situations, stress can be adequately handled through psychological behaviours and defences^16^. Naturally, such defence abilities are not the same for everybody, but depend on a complex range of factors, such as, experience, age, and possible psychological or physical problems. As a consequence, we can observe different defence styles, more or less adaptive, which may influence the maintenance of a state of relative wellness, or determine the loss of such an equilibrium causing the onset of a physical or psychical pathology^17^. In non-alexithymic individuals, either adequate or inadequate behavioural and psychological defences are activated to face a stressful event. However, when those stress factors are no longer mentally tolerable, more archaic psychological and biological defences appear, occupying the entire organism either globally or locally, in every single organ or apparatus^18^.

Quality of life in patients affected by IBD is an area of great interest for clinicians and researchers. Many surveys have deepened such field in patients affected by IBD, using adequate tools and markers^19^. A crucial factor in the reduction of the quality of life in IBD patients is the disease itself: inducing and maintaining remission for a long period of time are fundamental goals to gain improvements. Though, it clearly emerged in the literature that severity and activity of the disease are not the only factors predicting a reduction in patients’ quality of life^20^. Together with worries about (i) the loss or reduction of control of intestinal functions, (ii) the possibility of medical surgery, and (iii) side effects of pharmaceutical therapies, IBD patients also face repercussions on the body image of the self, and, more generally, changes in their lifestyle, because of new needs imposed by the disease. Moreover, psychosocial factors, not only influence the predisposition to the disease, but also affect the possibility of patients to functionally adapt to the pathological condition^21^,^22^.

In our work, we explore the association between personality traits such as psychoticism, extraversion, neuroticism, impulsivity, audacity and empathy, alexithymia, lifestyles, defence mechanisms such as turning against the object, projection, principalization, turning against the self and reversal and IBD, and we evaluate the eventual role played by them in relation to the pathogenesis of such disorders. The approach we want to pursue in this paper is slightly different from what has been done in the literature. Specifically, we shall identify the main determinants of IBD by investigating the features shared by patients, as they emerge from the answers to a questionnaire composed by several psychological tests. For this reason, we believe that an approach where tools and methodologies of Network Theory are used is most appropriate. In particular, we introduce a network representation of the system where the survey is considered as a bipartite network in which elements of one set are the participants, while elements of the other set are the answers they provide in the questionnaire. This would allow us to explore the connections amongst patients by simply performing a one-mode projection of the bipartite network, as detailed in the methods section. The structure of such connections will be used to characterize the relationships between IBD and psychological features. Moreover, our methodology is also able to select those connections amongst patients that are not compatible with a null hypothesis of randomness that takes into account the heterogeneity present in the system. We therefore have a statistically grounded way to select only the informative connections. We then (i) investigate what the level of similarity is between any two patients in our dataset, (ii) check whether or not such a similarity leads to the emergence of communities of patients characterized by specific features, and (iii) assess to which extent such features relate to IBD.

Although there is a growing body of literature on networks and their use in psychology^23^,^24^,^25^, our approach is rather new in the field. Indeed, following the approach originally introduced in ref. 26, there are two key aspects we want to emphasize as novelties of our approach: (i) the fact that we look at a survey as a bipartite network, and (ii) the fact that only statistically significant similarities between respondents are selected.

## Methods

We have analyzed the results of a survey of N = 100 people affected by chronic inflammatory intestinal disease who have been patients at the U.O.C. of Gastroenterology, Policlinico P. Giaccone, Palermo, Italy, in a time period of 19 months, ranging from May 2011 to December 2012. Patients are aged between 18 and 65 years old, and are affected by Ulcerative Colitis (UC) or Crohn’s Disease (CD), according to a diagnosis of IBD based on the classical clinical, radiological, endoscopic and histological criteria issued by the European Crohn’s and Colitis Organization (ECCO)^27^,^28^.

Patients affected by a severe intellectual disability, terminal illness, deafness and/or total blindness and/or aphasia have been excluded from the survey. The patients have been recruited with a diagnosis of chronic IBD and monitored by the Department of General and Specialistic Biomedicine of the Gastroenterology and Hepathology Ward of the University Hospital “P. Giaccone” in Palermo. Overall we had 55 patients affected by CD and 45 patients affected by UC. The case group is globally made of 51 male participants (with an average age of 40.7 years and a standard deviation of 17.3 years) and 49 female participants (with an average age of 40 years and a standard deviation of 14.1 years). The survey has also been administered to a control group of C = 66 people. The control group includes 66 participants with negative hystory of IBD and negative history of other psychological disorders and no record of previous hospitalization in any psychiatric unit. The control group is composed by 26 male participants (with an average age of 43.8 years and a standard deviation of 12.2 years) and 40 females (with an average age of 41.8 years and a standard deviation of 14.8 years).

The one presented here is a control-case observational study where all activities were performed according to guidelines approved by the Ethic Committee of the University Hospital “Paolo Giaccone” of the Palermo University (prot. n. 4/2011 on 13/04/2011). The study protocol was approved by the Ethic Committee of the University Hospital of the Palermo University (prot. n. 4/2011 on 13/04/2011).

Before testing, all the patients and controls gave their written informed consent, as required by Italian legislation – Law n.675, 12/31/1996.

### Psychological tests

The study involved the filling of clinical and anamnestic forms and use of the following tests: the Toronto Alexithymia Scale (TAS-20), the Eysenck Personality Questionnaire – Revised Brief Version (EPQ –R- Brief Version), the Eysenck Impulsivity Inventory (IVE), the 36-item Short Form Health Survey (SF-36) and the Defence Mechanisms Inventory (DMI). The psychometric properties of the Italian versions of all tests are reported in Appendix 4.

The Toronto Alexithymia Scale^29^ is a self-report questionnaire of 20 items based on a 5 point-Likert Scale. Every item represents a statement in relation to which a participant has to indicate his/her degree of accordance. The test is designed to provide an overall scale ranging between 20 and 100 that allows to distinguish between “non-alexithymic” (total score lower than or equal to 50), “borderline” (total score between 51 and 60), and “alexithymic” participants (total score larger than 60). The test is devised not only to calculate total scores, but also to associate a score with each one of the three dimensions that define the construct of Alexithymia: difficulty in identifying feelings (F1), difficulty in communicating feelings to others (F2), “Operatory Thinking”, or external thinking (F3).

The Eysenck Personality Questionnaire – Revised – Brief Version^30^ test is a 48, dichotomic (yes or no)- item questionnaire, divided into in 4 scales: Psychoticism, Extraversion, Neuroticism, Lie. The first three dimensions, according to Eysenck’s construct, represent the basic dimensions of personality; the fourth one is, instead, a control scale, aiming to reveal someone’s attempt to lie, through a distorted self evaluation. The typical extrovert person is sociable, with many friends, needs to meet people to talk with and does not like to be alone, is a stimulus seeker, takes any chance, loves changes, is carefree, uninhibited, optimistic, prefers to be always on the go and do many things, is generally an impulsive type, does not strictly control his feelings, and tends to be aggressive. Somebody who has a high score in Neuroticism, is anxious, worried about himself, moody, often depressed, shows sleep-wake rhythm disorders, suffers from many psychosomatic disorders, is very emotive, and his abnormal emotional reactions interfere with his capacity of adaptation, reacting irrationally and, sometimes, rigidly. High scores in Psychoticism describe someone who is solitary, not interested to others, scarcely adapting, lacking feelings and empathy, insensitive, hostile and aggressive even towards the loved ones, and defiant. The terms “Psychoticism” and “Neuroticism” do not have any pathological connotation, though show personality variables underlying “normal” behaviour. For each scale, the test has an average value of 50. Scores of 10 points above or below average are considered to indicate statistical significant deviations from the general population. In other words, values between 40 and 60 are usually associated to normal individuals.

The Eysenck Impulsivity Inventory^31^ is a 54, dichotomic (yes or no)-item inventory, divided into three scales: Impulsivity, Audacity, Empathy. IVE is used for purposes of research in many European and Extra-European countries for its extreme practicalness and possibility to obtain independent scores for each of the three scales constituting it. Impulsive participants can be defined as those who do not care and strongly underestimate the consequences of their actions, while audacious people are those who dare to risk, though well aware of the kind of risk they are taking on. Impulsivity is positively related to Psychoticism, Audacity is positively related to Extroversion and Empathy is positively related to Neuroticism, while negatively to Psychoticism. The same cut-offs as above apply to the IVE test.

The 36-item Short Form Health Survey^32^ test is a self-report questionnaire related to health state and quality of life. The perception of quality of life has a significant role in people with chronic pathologies such as IBD. The questionnaire was developed during the Eighties in the USA and successively translated and adapted to Italian culture. There are 36 questions forming 8 different scales whose validity has been largely documented: Physical Functioning (PF) - 10 questions, Role Physical (RP - physical limitations because of health state) - 4 questions, Role Emotional (RE), role limitations because of emotional state - 3 questions, Body Pain (BP) - 2 questions, General Health (GH), perception of general health status - 5 questions, Vitality (VT) - 4 questions, Social Functioning (SF) - 2 questions, Mental Health (MH) - 5 questions, Health Transitions (HT), changing in lifestyle - 1 question. All the questions in SF-36 but one refer to a period of time of 4 weeks before the filling of the questionnaire. SF-36 gives us one total and two partial scores, one about physical health and one about emotional health. After collecting the data, the attribution of scores to each question takes place in three phases: (i) recoding of the questions for the ten question associated to PF scale, as prescribed in ref. 32, (ii) calculation of the scales scores by summing the scores of the answers to the questions associated to the scales (raw scores of the scales), (iii) conversion of such raw scores of Physical health and Mental Health in scores ranging from 0 to 100, where 0 indicates the worst level and 100 the best level of functioning.

The Defence Mechanism Inventory^33^ test is a standardized tool, which evaluates defence mechanisms. They are classified in five defensive styles:

Turning Against Object (TAO): it is about defence mechanisms that block the relation to external world, intimacy, involvement, mature dependency, if used inadequately. Individuals predominantly using this defensive style are not able to analyse consequences of their actions and others’ reasons and emotions. They show a hot temper, hostility and vengeance underlying gruff and brutal relational behaviours. This defensive style aims to handle external threatens and hide very painful internal conflicts, creating an illusory sense of power, strength, dominion and control.

Projection (PRO): this defensive style involves projections and attribution of undesirable thoughts or emotions into other people. Affectivity is often characterized by disgust and resentment. External world is perceived as hostile and threatening as much as behaviours and impulses related to it. This style accomplishes three results at once: to get rid of one’s unacceptable characteristics; placing them into others and thus controlling them; creating justifications for behaviours and actions.

Principalization (PRN): this style involves more mature defences through which separate emotional from cognitive aspects to handle more easily the last ones with intellectual control. Such individuals accept loss and difficulties, even resigning to the hardest aspects of reality.

Turning Against Self (TAS): this style involves self -punishment. It aims to reduce threatens towards self-esteem foreseeing the worst and falsifying reality. Such mechanism creates a qualitative gap between judgement of the present and representation of the future. Affectivity is characterized by shame and despair, related to low self-esteem. Judgement of situations is biased by a self-punishing mental behaviour.

Reversal (REV): such defences minimize gravity of internal and external threatens misjudging them. Reality is distorted and the unacceptable parts of it are minimized, denied or ignored. Such individuals are usually polite, reliable and optimistic.

This test has a set of 12 stories to assess predominant or habitual defence styles. Each story has four levels of analysis: overt behaviours, phantasies, thoughts and emotions. For each defensive style the highest score you can get is 80, the cut-off under which we consider the use of a certain defensive style as pathological is a score of 70, a score of 65 is considered high, while one of between 40 and 60 can be considered normal.

### Data analysis

In order to assess what are the main psychological determinants of IBD we decided to use a network representation of the system where the survey is considered as a bipartite network in which elements of one set are the participants, while elements of the other set are the answers they provide in the questionnaire. Our approach will be articulated in three main steps: (*i*) to investigate what is the level of similarity between any two patients in our dataset (*ii*) to select those similarities that are not compatible with a null hypothesis of randomness that takes into account the heterogeneity present in the system, (*iii*) to check whether these similarities lead to the emergence of communities of homogeneous patients characterized by a certain feature and (*iv*) to assess to which extent such features are related to alexithymia and other psychological factors.

The statistical method used in our approach to assess the degree of similarity among the participants requires that survey results be described in terms of categorical variables^26^. Our methodology is therefore perfectly appropriate for the system we are considering, due to the fact that the answers given to the questionnaire are already in a categorical form. However, the method may suffer from coarse-graining effects. In other words, when the number of participants who give a certain answer to a question is too small, the statistical reliability of the similarity measure thus obtained is low, given that the confidence interval associated to the observed similarity value become too high. Therefore, we have modified the original database in order to remove possible sources of such a problem by aggregating together some answers. A detailed description of the aggregation process can be found in Appendix A3. After the aggregation of answers was completed we proceeded through further steps: network construction and characterization of communities.

### Network construction

A bipartite complex system is a system where one can distinguish two types of elements. The elements can be therefore gathered into two sets, namely set A and set B. Elements of set A are connected to elements of set B, but no explicit connection is present among the elements of set A and among the elements of set B. Examples include movies and actors^34^,^35^,^36^, authors and scientific papers^37^,^38^,^39^,^40^, email accounts and emails^41^, plants and animals that pollinate them^42^,^43^, criminals and crimes^44^, investors and trading days^45^, mobile phone users and calls^46^. A bipartite complex system can be represented by a bipartite network, i.e. a graph where nodes are grouped in two sets, say A and B, and links can only occur between elements of set A and elements of set B, that is, connections between elements of the same set are forbidden.

A projected network of set A is obtained by linking together those elements of set A that share at least one common first-neighbor element of B in the original bipartite system. This is a weighted network, where the weight, *w*_*ij*_, of a link between elements *i* and *j*, is the number of elements of set B that are first-neighbors of both *i* and *j* in the bipartite system. A statistically validated projected network of set A is obtained in two steps. First, a p-value is associated with each link in the projected network, representing the probability to observe a weight larger or equal to the observed one under a null hypothesis of random pairing. Then only links with a p-value lower than a given threshold of statistical significance — 1% corrected for multiple hypothesis testing according to the False Discovery Rate (FDR), in the present study — are selected. The method to construct a statistically validated network is detailed in ref. 47.

**Figure 1:**
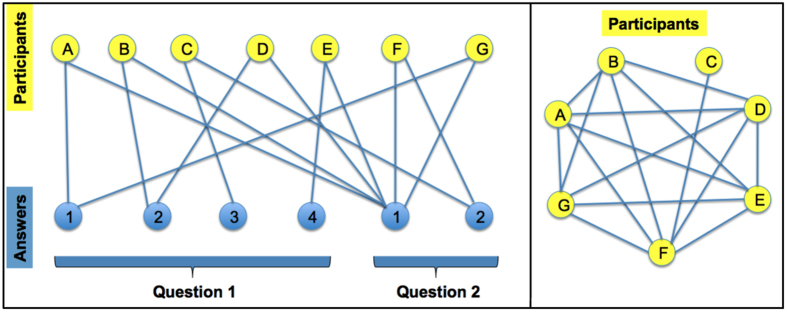
The left panel shows a schematic representation of the bipartite network of participants and their answers. We consider a set of 7 participants and 2 questions. The panel on the right shows a schematic representation of the resulting projected network on the side of Participants. For example, one can notice that participant C is connected only to participant F through Answer 2 to Question 2.

We believe that the system we are considering here can naturally be represented as a bipartite network where the two sets are given by the participants on one side and the answers they give to the questionnaire on the other side, as illustrated in Fig. 1 (left panel). In fact, no *a priori* connection exists between patients as well as no *a priori* association exists between the set of possible answers to the questionnaire (see ref. 26). Such a bipartite netwrok is further constrained by the fact that each participant gives one and only one answer to each question. This constraint requires one to suitably modify the null hypothesis considered in ref. 47, in order to apply the method of statistically validated networks to surveys, as shown in ref. 26 (see also ref. 48). Starting from the bipartite network one can construct a one-mode projection onto the set of participants as illustrated in Fig. 1 (right panel), by setting a link between two respondents if they provided the same answer to at least one question. The wieght of such a link is provided by the total number of answers that the pair of respondents have in common. Such a weight is then statistically validated, against a null hypothesis of random answer to each question, null hypothesis that takes into account the heterogeneity of answers, that is, the degree of “*popularity*” of possible anwers, to each question.

### Community detection and characterization

Given a network, i.e. a set of nodes (or vertices) connected by links, one can search for communities within the network. Heuristically, communities are subsets of nodes (people in our case) that display a higher level of connectivity among them with respect to their connectivity with other nodes in the network (see ref. 49 for a detailed discussion).

In this article, we consider different algorithms of community detection on networks. Specifically, we consider Infomap^50^, and the maximization of the modularity based algorithm Radatool^51^.

We characterize the resulting communities in terms of some psychiatric or sociological feature associated to the participants belonging to them. Specifically we use a statistical methodology illustrated in ref. 52, which essentially consists of a repeated construction of contingency tables and the application of Fisher’s exact test^53^ to examine the significance of the association in each table. Given the participants in a community and some attributes, like the gender, associated with each participant in the system, one can assess what are the attributes that are over-represented in the community, with respect to a null hypothesis of random sampling. The idea is that these attributes are those characterizing the community.

To characterize communities we use a 5% p-value threshold and the Bonferroni correction for multiple comparisons.

## Results

In this section, we summarize the results obtained by analyzing the adjacency one-mode projected network, which we call *original projected network*, and the associated statistically validated network. In this analysis, all the participants to the survey, both patients and controls, are included. Specifically, results about the original projected network of all participants and results obtained from the analysis of the associated statistically validated network (SVN) are separately discussed in the next two sections. The reason for considering these networks, and the corresponding communities of participants, separately is that they carry partially different information about the similarity structure of the system. The original projected network is a complete network, where the information about the similarity structure is only encoded in link weights. Therefore, some properties of the system can be hidden by the fact that many observed similarities might only appear by chance. As a consequence, communities are usually large and community detection algorithms may not reveal smaller emergent structures. On the other hand, the statistically validated network, which is the core of our methodology, only displays the structure of the system that emerges according to the strongest similarities among participants, and disregard weak similarities. As a result, in this network, communities are usually small and formed by very similar participants that likely will display a characterizing attribute. However, such a neat picture of homogeneous groups of participants comes at the price of loosing information about weaker similarities. Some participants may not appear at all in the statistically validated network, because they are not similar enough to any other participant. In summary, the analysis of the original projected network complements the analysis of the statistically validated network and vice versa.

### Original projected network of participants

The dataset we consider here is composed by 166 participants to the questionnaire as modified according to the procedure described in Appendix A3. There are 100 patients affected by UC disease (45 patients) and CD (55 patients) and 66 healthy individuals that play the role of control panel.

As illustrated in section “Methods”, we first constructed the adjacency projected network of the 166 participants. This is a network with 166 nodes and 13695 links. Such network has been partitioned by using the Radatool package thus obtaining two large communities R1 and R2 of 86 and 80 participants, respectively. This indicates that patients and controls are mixed up in the two communities. In fact, the R1 community contains 47 out of 66 control participants, 15 out 55 patients affected by Crohn disease and 24 out 45 patients affected by UC disease. The R2 community contains 19 out of 66 control participants, 40 out 55 patients affected by Crohn disease and 21 out 45 patients affected by UC disease.

By using the methodology of ref. 52, illustrated in section “Methods”, the two communities R1 and R2 can be characterized in terms of some psychiatric or sociological features associated to the participants included in each community. In a first investigation we tested the 41 features listed in Appendix A2. These features are a selection of the questionnaire questions. We assigned to each of the 166 participants an attribute for each of the 41 features, according to the rules illustrated in Appendix A1. A direct inspection of the detected over-expressions, reported in Table 1, let us conclude that the first community R1 of 86 elements is characterized by participants not affected by alexithymia, while the second community R2 composed of 80 participants involves the alexithymic ones.

As we mentioned above, the R1 community involves 47 control participants and 39 patients. The average TAS-20 score is 41.5 ± 9.6. Only one participant in R1 has a TAS-20 score larger than 60. The average TAS-20 score for the 47 control participants is 43.2 ± 8.4 while for the 39 patients is 40.0 ± 10.3. When considering the R2 community, we have an overall average TAS-20 score of 53.2 ± 12.9 and 25 participants have a TAS-20 score larger than 60. The average TAS-20 score for the 19 control participants is 53.5 ± 15.0 while for the 61 patients is 53.1 ± 12.3. The TAS-20 score is higher in the R2 community than in the R1, in line with the community characterization analysis. However, the differences between the TAS-20 score for patients and control participants is quite small in the two communities. This is in line with the fact that the community detection algorithm groups together participants with a similar profile.

As a counter-check, in a second investigation we wanted to verify whether or not the two communities were mainly composed of patients or control participants. Therefore we considered being patients or control as a new feature and used the attributes “patient” or “control” accordingly. Our analysis shows that for the community R1 the attribute “control” results to be overexpressed, while the attribute “patient” is over-expressed for the R2 community.

### Statistically validated network of participants

In a second investigation we considered the same dataset as above and constructed the statistically validated projected network of the 166 patients. This is a network with 128 nodes and 1066 links. The validated network involves 56 control cases, 32 patients affected by Crohn disease and 40 patients affected by the UC disease.

Also in this case, the network has been partitioned by using the Radatool package thus obtaining four communities R1, R2, R3 and R4 of 49, 35, 25 and 19 participants, respectively. The S1 community contains 20 control participants, 11 patients affected by Crohn disease and 18 patients affected by UC disease. The S2 community contains 22 control participants, 5 patients affected by Crohn disease and 8 patients affected by UC disease. The S3 community contains 5 control participants, 11 patients affected by Crohn disease and 9 patients affected by UC disease. The S4 community contains 9 control participants, 5 patients affected by Crohn disease and 5 patients affected by UC disease.

The characterization of these communities has been done by using the 41 features listed in Appendix A2, as in the previous section. A direct inspection of the detected over-expressions, reported in Table 2, let us conclude that in the community S3 of 25 participants the attribute “Alexithymic” is overexpressed together with the female attribute, while in S1 of 49 participants the attribute “Borderline” is overexpressed. In the other two communities we have non alexithymic patients with low level of neuroticism in S2 and male non alexithymic patients with high levels of audacity in S4.

As mentioned above, the SVN allows for a finer analysis of the set of participants. In particular, the large community R1 of Table 1 splits into the four communities of Table 2: 28 out of 86 participants are now present in S1, 35 out of 86 participants are now present in S2, 2 out of 86 participants are now present in S3, and 17 out of 86 participants are now present in S4. It is remarkable the fact that only two participants from R1, involving non alexithymic participants, are found in S3, which is the only community where the attribute “Alexithymic” is overexpressed. On the other side, when considering the large community R2 of Table 1 we observe that 21 out of its 80 participants are now present in S1, 23 out of its 80 participants are now present in S3, and 2 out of its 80 participants are now present in S4. Again the R2 community, involving alexithymic participants, essentially splits into the two community of the validated network characterized by the attribute alexithymic and borderline. That shows that indeed the statistically validated network can be seen as a magnifying lens that allows to study the communities of the original network in a more detailed way.

## Discussion

**Figure 2:**
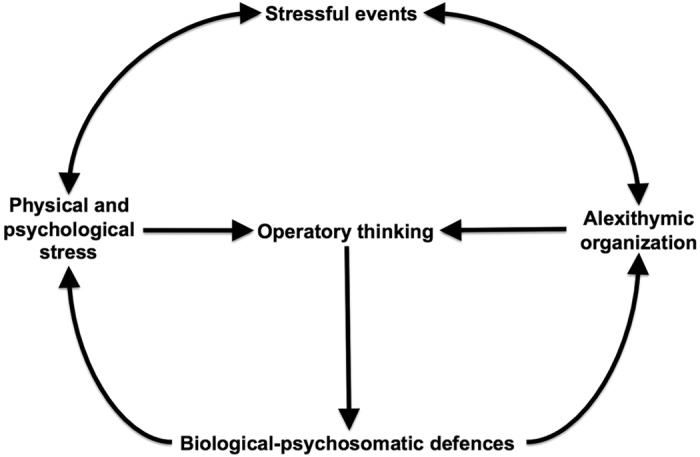
Response to stressful conditions in alexithymic patients.

Most of the over-expressed attributes are associated to low levels of health and quality of life. Globally, after considering both cases and controls, it has been found a correlation between low levels of physical and mental health and diagnosis of alexithymia, as measured by TAS-20, high levels of the neuroticism dimension, as measured by EPQ-R, and elevated impulsivity. As far as “Alexithymia” is concerned, we noticed a more relevant role of the third factor considered in the TAS-20 test which considers a specific feature of emotional functioning called “external thinking” indicating a personality disposition inclined to solving internal conflicts by their external projection through actions. Such a concept, illustrated in Fig. 2, was originally defined by Trombini–Baldoni, see figure 9.1 of ref. 54.

Alloplastic modifications can be interpreted as changes in the emotional organization centered on the external origin of stress, namely, on the event as such. Typical situations are: the individual attempts to modify reality, e.g., by facing conflicts at work or quitting the job. Autoplastic modifications, on the other hand, can be described as changes of the emotional organization centered on the regulation of emotions, in order to face the emotional unease aroused by stress by means of more or less evolved and adaptive defense mechanisms, or by means of psychical working-through, leading to a psychical solution to the problem. These strategies are only accessible to people who do not have an alexithymic organization^55^. The appearance of somatic symptoms, which gradually tend to replace emotions, determines a “psycho-somatization of affects” that impoverishes emotional life and can be considered as the expression of an incomplete development of emotional psychological capabilities. This observations is particularly important because of its direct correlation with the impulsivity dimension, so that the less a participant uses abstract, symbolic and emotional thinking, the more he will tend to use defensive mechanisms focused on action, not only with an impulsive significance but also with an obsessive-compulsive, ritualistic or even adaptive attribution. Referring to our sample, by the way, this trait seems to be firmly correlated with an highly expressed impulsive personality trait, that usually carries a maladaptive valence and it is, indeed, associated with a worst state of illness and global lower quality of life.

One more remark concerns the gender profile of the participants. The pattern of features characterized by high neuroticism, diagnosis of alexithymia (particularly with reference to the third factor of TAS-20), high impulsivity and the correlation with more severe physical conditions, due to both CD and UC, (i.e. low levels of physical health as in S3 or Crohn disease as in R2) seems to be more characteristic of female gender. In fact, both in S3 and R2 the female attribute has been statistically validated at a 5% univariate p-value threshold with Bonferroni correction for multiple comparison. On the other hand, some more specifically masculine features could have a protective role on the development of particular internistic diseases. In fact, in the assessed groups male people seemed to overcome “Alexithymia” by means of elevated “Audacity” associated to low “Neuroticism”, see communities R1 and S4. This data can be explained by considering that it is usually supposed that there exists a gender specific psychological configuration according to which females have a stronger disposition to introspective dimension, inhibition of action and more reactive affectivity, while males are more often predisposed to external exploration, extroversion, facilitated actions over affectivity as a reaction to conflicts. We recall that, according to its definition, audacity is the tendency to face the danger with all the risks that such a tendency implies. The consequence is an increased feeling of physical well being and vitality. Therefore, that would indicate that audacious participants show an improved capacity of envisioning and taking adaptive strategies characterized by heteroplastic changes in the emotional experience^56^. These findings induce to the formulation of further hypothesis such as the possibility that in a population of alexithymic participants females should be more often affected by psychosomatic diseases, while male participants should easier develop behavioral disorders (i.e. drug addiction). However, many studies on the distribution of alexithymia in a normal population show that there is a significant difference between males and females, and in particular that males are more alexithymic than females^57^,^58^,^59^,^60^,^61^. In agreement with these findings, our results suggest that alexithymic women are more likely to develop IBD than alexithymic men that tend to externalize their inner discomfort with actions or behaviors. Literature data show that even if there are no significant difference between genders in IBD the male-female ratio for subjects affected by Crohn’s disease ranges from 1:1.8 to even 1:2. The overall female incidence rate appears to be about 1,5 times higher than in men. It is known that females and males with IBD can differ in some psychosocial aspects of disease and that psychological morbidity has a contributory role most notably in females. Quality of life is reduced in females compared with males with IBD^62^. Psychological factors play a greater role in some females, with greater disease-related concerns and worries about being a burden or being treated differently. They also rate their symptoms as being more severe and more extensive disease may be commoner in females. However, even in the general population, self-rated quality of life scores tend to be lower in females than males^63^.

Finally, it’s worth discussing here some caveats and limitations of the presented statistical analysis. First of all, our results are based on a cross-sectional statistical analysis of data, which allowed us to single out statistically significant similarities between the profiles of patients and controls. Therefore, though our analysis demonstrates the existence of a statistically significant association between psychosomatic elements and the development of Chronic Inflammatory Bowel Diseases, we cannot conclude that a causal relationship between the two exists.

Another limitation of the present analysis is that different items of the questionnaire are assumed to be logically independent in the null hypothesis used to test the similarity between patients’ profiles. Therefore logically dependent questions (and the corresponding answers) have been pre-processed and aggregated in a single question, in order to obtain a modified database only including responses to logically independent questions.

On the other hand, we believe that our approach has significant advantages. Specifically, the network approach we pursued to describe the system allowed us to obtain a global vision of the system that fully takes into account the heterogeneity of the individuals participating in the study, as detected by the psychological tests. Moreover, the fact that each link present in the network(s) is statistically validated, and indicates a degree of similarity between two participants’ profiles that is extremely unlikely to be observed by chance, naturally provides a list of selected relationships that are most relevant to describe the structure of the considered multivariate system, e.g., by distinguishing between communities of participants with more homogeneous profiles.

## Conclusions

In conclusion, according to classic psychiatric literature and also based on the results of this study, we think it is possible to determine a clear and marked influence of psychosomatic elements on the onset, and moreover on the development and repercussions on quality of life of chronic IBD. We also identified a group of participants particularly vulnerable to the development of IBD characterized by psychosomatic influences and this class is made by participants with high expression of neuroticism, alexithymic (especially external thinking) and impulsive traits, possibly of female gender. Although this study has shown the importance of a psychological functioning specific element as a predictor of a psychosomatic conformation, we could not demonstrate the existence of a causality relationship between the psychosomatic disposition and the clinical manifestation of IBD.

More generally, from a methodological point of view, we have shown how tools and methodologies of network theory and multivariate analysis can be fruitfully used to reveal the structure and properties of a real-world multilayer system, which includes sociological, clinical, and biomedical information. In this respect, we believe we are positively contributing to show how useful it can be to apply quantitative methods of complex system analysis within the context of the psychiatric discipline.

## Additional Information

**How to cite this article**: La Barbera, D. *et al*. Alexithymia and personality traits of patients with inflammatory bowel disease. *Sci. Rep.* **7**, 41786; doi: 10.1038/srep41786 (2017).

**Publisher's note:** Springer Nature remains neutral with regard to jurisdictional claims in published maps and institutional affiliations.

## Supplementary Material

Supplementary Information

## Figures and Tables

**Table 1:** OVER_expressed attributes for the dataset described in section 3.1.

Community R_i_	Size	FEATURE	ATTRIBUTE A_j_	n_i_	n_ij_	p-value
R1	86	SF 36	*Normal*	104	80	0
R1	86	Sf 36 Physical health	*Normal*	98	74	0
R1	86	SF 36 Mental health	*Normal*	79	68	0
R1	86	TAS- 20 F1	*Normal*	146	86	1.12E-07
R1	86	TAS diagnosis	*Normal*	99	67	5.50E-07
R1	86	TAS- 20 F2	*Normal*	144	85	6.21E-07
R1	86	TAS- 20 F3	*Normal*	145	85	1.43E-06
R1	86	EPQ R neuroticism	*Low*	36	30	1.20E-05
R1	86	Diagnosis	*n.a.*	66	47	3.96E-05
R1	86	Partial Mayo	*n.a.*	66	47	3.96E-05
R1	86	HBI	*n.a.*	66	47	3.96E-05
R1	86	IBD severity	*n.a.*	66	47	3.96E-05
R1	86	Pattern CD	*n.a.*	66	47	3.96E-05
R1	86	Montreal CD	*n.a.*	66	47	3.96E-05
R1	86	Montreal UC	*n.a.*	66	47	3.96E-05
R1	86	Type of therapy	*n.a.*	66	47	3.96E-05
R1	86	Biological therapy	*n.a.*	66	47	3.96E-05
R1	86	IBD genetic predisposition	−*99*	66	46	1.47E-04
R1	86	Use of tobacco	−*99*	46	34	3.33 E-04
R1	86	Partner job	−*99*	103	63	1.66 E-03
R1	86	Sex	*M*	77	49	3.36E-03
R1	86	Partner working hours	−*99*	124	72	4.59E-03
R1	86	IVE Impulsivity	*Low*	17	14	6.68E-03
R2	80	SF36	*Low*	62	56	0
R2	80	SF36 Physical health	*Low*	68	56	0
R2	80	SF36 Mental health	*Low*	87	69	0
R2	80	TAS-20 diagnosis	*Alexithymic*	26	25	1.93E-08
R2	80	EPQ R Neuroticism	*High*	22	21	6.21E-07
R2	80	TAS-20 F1	*F1*-*F3*	18	18	6.46E-07
R2	80	IBD severity	*Yes*	51	38	5.23E-06
R2	80	Montreal UC	−*99*	53	39	6.23E-06
R2	80	Diagnosis	*Crohn*	55	40	7.25E-06
R2	80	Partial Mayo	−*99*	55	40	7.25E-06
R2	80	Montreal CD	*Ileocolic*	35	28	1.79E-05
R2	80	TAS-20 F2	*F2*-*F3*	16	15	7.93E-05
R2	80	TAS-20 F3	*F2*-*F3*	16	15	7.93E-05
R2	80	Partner job	*Employee*	30	23	4.93E-04
R2	80	Type of therapy	*Biological*	36	26	9.67E-04
R2	80	Biological therapy	*Yes*	36	26	9.97E-04
R2	80	IVE Empathy	*High*	28	21	1.63E-03
R2	80	Sex	*F*	89	52	3.56E-03
R2	80	Partner working hours	*full – time*	31	22	4.21E-03

We used the Bonferroni correction for multiple comparison starting from un 5% univariate p-value threshold. Null values indicate a p-value smaller than 10^−8^.

**Table 2:** OVER_expressed attributes for the dataset described in section 3.2.

Community S_i_	Size	FEATURE	ATTRIBUTE A_j_	n_i_	n_ij_	p-value
S1	49	Education	*primary school*	12	10	0
S1	49	Job	*Retired*	13	11	0
S1	49	TAS diagnosis	*Borderline*	28	19	0
S1	49	IVE Audacity	*Low*	44	30	6 E-7
S1	49	EPQ R Lie	*High*	49	31	5 E-6
S1	49	Age	*5*	24	19	7 E-6
S2	35	Partner job	−*99*	82	30	0
S2	35	Tobacco	−*99*	39	18	0
S2	35	TAS-20 diagnosis	*Normal*	87	31	0
S2	35	SF36	*Normal*	92	33	0
S2	35	EPQ R S Neuroticism	*Low*	34	21	5 E-7
S2	35	SF36 Mental health	*Normal*	74	30	5 E-5
S3	25	Sex	*F*	69	20	0
S3	25	Montreal UC	−*99*	30	12	0
S3	25	TAS -20 F1	*F1*-*F3*	12	8	0
S3	25	TAS-20 F2	*F2*-*F3*	9	6	0
S3	25	TAS-20 F3	*F2*-*F3*	9	6	0
S3	25	SF 36	*Low*	36	21	0
S3	25	IVE Impulsivity	*High*	13	8	0
S3	25	IVE Empathy	*High*	24	11	0
S3	25	SF 36 Physical health	*Low*	40	20	2 E-8
S3	25	SF 36 Mental health	*Low*	54	22	2 E-7
S3	25	TAS diagnosis	*Alexithymic*	13	9	4 E-5
S3	25	EPQ R SNeuroticism	*High*	13	9	4 E-5
S4	19	Sex	*M*	59	16	0
S4	19	TAS diagnosis	*Normal*	87	19	0
S4	19	DMI REV	*Normal*	93	19	0
S4	19	IVE Audacity	*High*	10	7	4 E-5

We used the Bonferroni correction for multiple comparison starting from un 5% univariate p-value threshold. Null values indicate a p-value smaller than 10^−8^.
