# Supplementary material for: Alexithymia and personality traits of patients with inflammatory bowel disease
Source: Sci Rep. 2017 Feb 2;7:41786. doi: 10.1038/srep41786 (PMC5288771; doi:10.1038/srep41786)
Supplement: Supplementary Information [file srep41786-s1.pdf]

Alexithymia and personality traits of patients with inflammatory bowel disease

D. La Barbera[1], B. Bonanno[1], M. V. Rumeo[1], V. Alabastro[1], M. Frenda[1], E. Massihnia[2], M.C. Morgante[3], L. Sideli[1], A. Craxì[4], M. Cappello[4], M. Tumminello[5], S. Micciché[6], L. Nastri[1]

[1]Department of Experimental Biomedicine and Clinical Neuroscience, Unit of Psychiatry, University Hospital, Palermo, Italy
[2]Unit of Nefrology II with Dialysis and Renal Transplantation, ARNAS Civico Di Cristina Benfratelli, Palermo
[3]Biomedical Department of Internal and Specialty Medicine, Regional Reference Center for Metabolism rare pathologies, University Hospital, Palermo, Italy
[4]Biomedical Department of Internal and Specialty Medicine, Unit of Gastroenterology, University Hospital, Palermo, Italy
[5]Department of Economics, Management and Statistics, University of Palermo, Palermo, Italy
[6]Department of Physics and Chemistry, University of Palermo, Palermo, Italy

APPENDIX A1: Construction of Attributes

Let us call $S_{i,f}$ the answer/score of the *i-th* participant to the question corresponding to the *f-th* feature, let us call $A_{i,f}$ the attribute of the *i-th* participant to the *f-th* feature. According to Ref. [29, 32, 33] we have:

1. For the features F1, F3, and from F4 to F23, the attribute was given by the answer/score given by the participant in the questionnaire: $A_{i,f}=S_{i,f}$.

2. For Feature F2 (Age) we simply computed the distribution of all ages of the 166 participants and categorized each participant's age in terms of the 5-quantiles of such distribution.

3. For the features from F27 to F36 we assigned to each participant three possible attributes according to the score $S_{i,f}$ present in the questionnaire and following the rule, according to Ref. [32]:

   a. $S_{i,f}>60$ A_{if}=HIGH

   b. $40<S_{i,f}<=60$ A_{if}=NORMAL

   c. $S_{i,f}<=40$ A_{if}=LOW

4. For the feature F24 (f=24) we assigned to each participant three possible attributes according to the score $S_{i,f}$ present in the questionnaire and following the rule, according to Ref. [29]:

   a. If the patient is alexythimic and $|S_{i,f}-S_{i,f+1}|>10$ and $|S_{i,f}-S_{i,f+2}|>10$ $A_{if}=F1$

   b. If the patient is alexythimic and $|S_{i,f}-S_{i,f+1}|>10$ $A_{if}=F1\text{-}F2$

   c. If the patient is alexythimic and $|S_{i,f}-S_{i,f+2}|>10$ $A_{if}=F1\text{-}F3$

   d. In all other cases we set $A_{if}=NORMAL$

5. For the feature F25 (f=25) we assigned to each participant three possible attributes according to the score $S_{i,f}$ present in the questionnaire and following the rule, according to Ref. [29]:

   a. If the patient is alexythimic and $|S_{i,f}-S_{i,f-1}|>10$ and $|S_{i,f}-S_{i,f+1}|>10$ $A_{if}=F2$

   b. If the patient is alexythimic and $|S_{i,f}-S_{i,f-1}|>10$ $A_{if}=F1\text{-}F2$

   c. If the patient is alexythimic and $|S_{i,f}-S_{i,f+1}|>10$ $A_{if}=F2\text{-}F3$

   d. In all other cases we set $A_{if}=NORMAL$

6. For the feature F26 (f=26) we assigned to each participant three possible attributes according to the score $S_{i,f}$ present in the questionnaire and following the rule, according to Ref. [29]:

   a. If the patient is alexythimic and $|S_{i,f}-S_{i,f-2}|>10$ and $|S_{i,f}-S_{i,f-1}|>10$ $A_{if}=F3$

   b. If the patient is alexythimic and $|S_{i,f}-S_{i,f-2}|>10$ $A_{if}=F1\text{-}F3$

   c. If the patient is alexythimic and $|S_{i,f}-S_{i,f-1}|>10$ $A_{if}=F2\text{-}F3$

   d. In all other cases we set $A_{if}=NORMAL$

7. For the feature F37 (f=37) we assigned to each participant four possible attributes according to the score $S_{i,f}$ present in the questionnaire and following the rule, according to Ref. [33]:

   a. $S_{i,f}>65$ and $S_{i,g}< S_{i,f}-6$ A_{if}=TAO

   The above condition must hold simultaneously for all features *g=F38, F39, F40, F41*

   b. $S_{i,f}>60$ and $S_{i,f+1}>60$ and $|S_{i,f+a}- S_{i,f}|<6$ and $S_{i,g}<60$ A_{if}=TAO/PRO

The above condition must hold simultaneously for all features $g=F39, F40, F41$

c. $S_{i,f}>60$ and $S_{i,f+3}>60$ and $|S_{i,f+a}- S_{i,f}|<6$ and $S_{i,g}<60$ A_{if}=TAO/TAS

The above condition must hold simultaneously for all features $g=F38, F39, F41$

d. In all other cases we set $A_{if}=NORMAL$

8. For the feature F38 (f=38) we assigned to each participant three possible attributes according to the score $S_{i,f}$ present in the questionnaire and following the rule, according to Ref. [33]:

a. $S_{i,f}>63$ and $S_{i,g}< S_{i,f}-7$ A_{if}=PRO

The above condition must hold simultaneously for all features $g=F37, F39, F40, F41$

b. $S_{i,f}>60$ and $S_{i,f+2}>60$ and $S_{i,g}<60$ A_{if}=PRO/TAS

The above condition must hold simultaneously for all features $g=F37, F39, F41$

c. In all other cases we set $A_{if}=NORMAL$

9. For the feature F39 (f=39) we assigned to each participant three possible attributes according to the score $S_{i,f}$ present in the questionnaire and following the rule, according to Ref. [33]:

a. $S_{i,f}>64$ and $S_{i,g}< S_{i,f}-6$ A_{if}=PRN

The above condition must hold simultaneously for all features $g=F37, F38, F40, F41$

b. $S_{i,f}>60$ and $S_{i,f+2}>60$ and $S_{i,g}<60$ A_{if}=PRN/REV

The above condition must hold simultaneously for all features $g=F37, F39, F41$

c. In all other cases we set $A_{if}=NORMAL$

10. For the feature F40 (f=40) we assigned to each participant three possible attributes according to the score $S_{i,f}$ present in the questionnaire and following the rule, according to Ref. [33]:

a. $S_{i,f}>65$ and $S_{i,g}< 30$ A_{if}=TAS

The above condition must hold simultaneously for all features $g=F37, F38, F39, F41$

b. $S_{i,f}>60$ and $S_{i,f+1}>60$ and $| S_{i,f} - S_{i,f+1}|$<6 and $S_{i,g}<60$ A_{if}=TAS/REV

The above condition must hold simultaneously for all features $g=F37, F38$

c. In all other cases we set $A_{if}=NORMAL$

11. For the feature F41 (f=41) we assigned to each participant two possible attributes according to the score $S_{i,f}$ present in the questionnaire and following the rule, according to Ref. [33]:

a. $S_{i,f}>65$ and $S_{i,g}< S_{i,f} -5$ A_{if}=REV

The above condition must hold simultaneously for all features $g=F37, F38, F39, F40$

b. In all other cases we set $A_{if}=NORMAL$

12. Finally we assigned the attribute -99 when no answer/score was given to the questionnaire

APPENDIX A2: List of Attributes

FEATURE	COLUMN in the database file	DENOMINATION
F1	C2	Sex
F2	C3	Age
F3	C4	Diagnosys
F4	C5	Partial Mayo
F5	C6	HBI
F6	C7	IBD severity
F7	C8	Pattern CD
F8	C9	Montreal CD
F9	C10	Montreal UC
F10	C11	Type of therapy
F11	C12	Biological therapy
F12	C13	Marital status
F13	C14	With whom you live
F14	C15	Education
F15	C16	Job
F16	C17	Working hours
F17	C18	Partner job
F18	C19	Partner working hours
F19	C20	Problems at work
F20	C21	Problem within family
F21	C22	IBD genetic predisposition
F22	C23	Tobacco
F23	C24	TAS-20 diagnosis
F24	C25	TAS -20 F1
F25	C26	TAS-20 F2
F26	C27	TAS-20 F3
F27	C28	SF 36
F28	C29	SF 36 Physical health
F29	C30	SF 36 Mental health
F30	C31	EPQR Psychoticism
F31	C32	EPQR Extroversion
F32	C33	EPQR Neuroticism
F33	C34	EPQR Lie
F34	C35	IVE Impulsivity
F35	C36	IVE Audacity
F36	C37	IVE Empathy,
F37	C38	DMI_TAO
F38	C39	DMI_PRO
F39	C40	DMI_PRN
F40	C41	DMI_TAS
F41	C42	DMI_REV”

APPENDIX A3: Database modifications

2.2.1 Aggregation of answers

1. **TAS-20 (all the 20 questions)** – the possible answers to this question have been grouped as follows (original answer – modified answer):
    a. Totally disagree, and partially disagree – answer 1
    b. Neutral – answer 2
    c. Totally agree, and partially agree – answer 3
2. **SF-36 (question 1)** – the possible answers to this question have been grouped as follows:
    a. 1,2,3 – answer 1
    b. 4 – answer 2
    c. 5 – answer 3
3. **SF-36 (question 2)** – the possible answers to this question have been grouped as follows:
    a. 1,2 – answer 1
    b. 3 – answer 2
    c. 4,5 – answer 3
4. **SF-36 (question 6)** – the possible answers to this question have been grouped as follows:
    a. 1 – answer 1
    b. 2,3 – answer 2
    c. 4,5 – answer 3
5. **SF-36 (question 7)** – the possible answers to this question have been grouped as follows:
    a. 1 – answer 1
    b. 2,3,4 – answer 2
    c. 5,6 – answer 3
6. **SF-36 (question 8)** – the possible answers to this question have been grouped as follows:
    a. 1 – answer 1
    b. 2,3 – answer 2
    c. 4,5 – answer 3
7. **SF-36 (question 9a to 9i)** – the possible answers to this question have been grouped as follows:
    a. 1,2 – answer 1
    b. 3,4 – answer 2
    c. 5,6 – answer 3
8. **SF-36 (question 10)** – the possible answers to this question have been grouped as follows:
    a. 1,2 – answer 1
    b. 3 – answer 2
    c. 4,5 – answer 3
9. **SF-36 (question 11a to 11d)** – the possible answers to this question have been grouped as follows:
    a. 1,2 – answer 1
    b. 3 – answer 2
    c. 4,5 – answer 3

APPENDIX A4: Psychometric properties of the Italian version of the considered tests

TAS-20

Related Bibliography

Craparo G, Faraci P, Gori A: Psychometric Properties of the 20-Item Toronto Alexithymia Scale in a Group of Italian Younger Adolescents, Psychiatry Investig. 2015, 12(4): 500–507.

In the above-mentioned paper, a sample of 508 Italian young people was studied in order to investigate the TAS-20 psychometric properties. The Reliability coefficients, i.e. the Cronbach's α values, are in the range from 0.40 (F3) to 0.69 (F1). The TAS-20 factors F1, F2 and F3 correlated moderately with each other ($-0.22 \leq r \leq 0.21$, with a p-value $p<0.01$), except for the correlation between factor F2 (Difficulty describing feelings) and factor F3 (Externally oriented thinking) ($r=-0.02$), that was non-significant at a 1% confidence level.

SF-36

Related Bibliography

Apolone G, Mosconi P, The Italian SF-36 Health Survey: Translation, Validation and Norming, J Clin Epidemiol 1998, 51 (11): 1025–1036.

Apolone G, Cifani S, Mosconi P. Questionario sullo stato di salute SF-36. Traduzione e validazione della versione italiana: Risultati del progetto IQOLA. Medic 1997; 2: 86–94

In the first study mentioned above, a sample of 2031 individuals was divided into different groups based on census data. The Cronbach's α values were in the range between 0.77 to 0.93 with the lowest values in the General Health (GH) scale and Social Functioning (SF) scale. There was more variation across groups (range from 0.55 to 0.94) than inside the groups. In general, internal consistency reliability was high, with most of the lowest values in the GH scale, the young age groups and the more educated samples.

The proportion of total variance in each scale explained by the two extracted components in the Italian data was 60% to 78% across scales, indicating that the two factors explained the majority of the variance in each scale. The ordering of correlations between the eight scales and both factors was generally equivalent in the United States and in Italy.

In the second study mentioned above, The Quality of Life Assessment (IQOLA) project tested the assumption that the SF-36, originally developed in the United States and probably the most widely used instrument in English-speaking countries, can be translated, validated, and normed in other languages while maintaining its excellent content and psychometric and clinical validity.

IVE

Related Bibliography

Russo PM, Leone L, De Pascalis V, Cross-cultural validity of the I_7 impulsiveness-venturesomeness-empathy scales: evidence from the Italian I_7, Comprehensive Psychiatry 2011, 52 (4): 446–452.

Barrett P, Petrides KV, Eysenck SBG, Eysenck HJ. The Eysenck Personality Questionnaire: an examination of the factorial similarity of P, E, N, and L across 34 countries. Pers Individ Diff 1998; 25: 805-819

In the study mentioned above, the authors presented the psychometric properties of the Italian I_7 focusing primarily on its structural similarity with other linguistic versions of the measure. The cross-cultural generalizability of Impulsiveness-Venturesomeness-Empathy questionnaire (I_7) was investigated with a sample of 578 Italian adults, mostly nonstudents. The reliability of I_7 was computed using the Kuder-Richardson 20 (KR-20) formula for measuring reliability of scales with dichotomous items. The

similarities in factor solutions across sexes and across the factorial structures observed in other languages were tested using the modified Kaiser-Hunka-Bianchini (KHB) index, the congruency coefficient index, and the double-scaled Euclidean similarity (DSES) index developed by Barrett et al, 1998. Congruency coefficients larger than 0.90 would indicate acceptable congruence between 2-factor solutions. The Cronbach's α values were 0.81 for Extroversion (E), 0.82 for Neuroticism (N), and 0.75 for the Psychoticism (P) scales. Reliability level was acceptable for impulsiveness (KR-20 = 0.78) and venturesomeness scales (KR-20 = 0.82) but barely sufficient for empathy scale (KR-20 = 0.70).
Factor similarity indexes of the Italian version with the original Eysenck scale revealed Kaiser-Hunka-Bianchini (KHB) and double-scaled Euclidean similarity (DSES) coefficients higher than 0.90 for impulsiveness and venturesomeness, but not for empathy (average KHB = 0.85, average DSES = 0.88).

EPQRS

Related Bibliography

San Martini P, Mazzotti E., Setaro S., Factor structure and psychometric features of the italian version for the EPQ-R, Personality and Individual Differences 1996, 21 (6): 877-882
Eysenck, S. B. G., Confronti transculturali tra le personalità di soggetti italiani continentali, siciliani e inglesi. Bollettino di Psicologia applicata 1985. 100: 11-16.

In the first reference mentioned above, factor structure and psychometric properties of the EPQ-R test were investigated in an Italian sample of 553 subjects (328 females and 225 males). The main psychometric features of the questionnaire were similar to those reported for the original English version. Factor analysis, however, showed six (out of 32) items of the Psychoticism (P) scale, referring chiefly to compliance to formal rules of conduct, to have their highest loadings on the Lie (L) factor. The consistency of this finding with previous results suggested that, in the Italian context, these items tend to be more closely connected with the social conformity aspect of the L scale than with the psychopathy facet of P and should be excluded from the scoring of the P dimension
The Cronbach's α values ranged from 0.70 P to 0.88 Neuroticism (N). The skewness for the considered females was similar in value to that reported for the British EPQ-R (0.95 vs 0.90, reported in Eysenck et al., 1985), and higher for males (1.53 vs 1.02 reported by Eysenck et al., 1985). Also the general pattern of the correlations between the scales was substantially unchanged in the revised form, including the excessive negative correlation between P and L. The internal consistency of the P scale was slightly higher.

DMI

Related Bibliography

Gleser GC, Ihilevich D, An objective instrument for measuring defense mechanisms, Journal of Consulting and Clinical Psychology 1969, 33 (1): 51-60

The Cronbach's α values ranged from 0.69 to 0.71 The product-moment correlations between the separate defence scores ranged from 0.85 for PRO to 0.93 for TAO. The average correlation was 0.89. The test stability was checked with a test-retest procedure. Assuming a 5% confidence level no significant temporal variation was observed in any of the DMI scales.
